# ADAPTIVE HORSEBACK RIDING: AN INNOVATIVE PROGRAM FOR IMPROVING THE QUALITY OF LIFE OF PERSONS LIVING WITH DEMENTIA

**DOI:** 10.1093/geroni/igac059.176

**Published:** 2022-12-20

**Authors:** Beth Fields

**Affiliations:** University of Wisconsin-Madison, Madison, Wisconsin, United States

## Abstract

More than 5 million Americans living with Alzheimer’s disease and related dementia (ADRD) have likely experienced forced social isolation due to the COVID-19 pandemic and subsequent stay-at-home mandates and public closures. Forced social isolation negatively impacts the quality of life of persons living with ADRD by worsening behavioral and psychological symptoms. Given continued pandemic restrictions and poor outcomes, now is the time to develop and support programs designed to improve quality of life through meaningful interactions that occur in natural environments. This symposium will introduce ‘Riding in the Moment’, an adaptive riding program that improves the quality of life of persons living with ADRD. Program participants are provided a safe, social, and supportive natural environment where they can ride, groom, and care for horses. The experiences constructed from Riding in the Moment seek to impact the current public health crisis by reducing social isolation for a growing and vulnerable population.

